# Balloon kyphoplasty in malignant spinal fractures: a systematic review and meta-analysis

**DOI:** 10.1186/1472-684X-8-12

**Published:** 2009-09-09

**Authors:** Carmen Bouza, Teresa López-Cuadrado, Patricia Cediel, Zuleika Saz-Parkinson, José María Amate

**Affiliations:** 1 Health-Care Technology Assessment Agency, Instituto de Salud Carlos III, Madrid, Spain

## Abstract

**Background:**

Spinal fractures are a common source of morbidity in cancer patients. Balloon Kyphoplasty (BKP) is a minimally invasive procedure designed to stabilize fractures and correct vertebral deformities. We performed a meta-analysis to determine the efficacy and safety of BKP for spinal fractures in cancer patients.

**Methods:**

We searched several electronic databases up to September 2008 and the reference lists of relevant publications for studies reporting on BKP in patients with spinal fractures secondary to osteolytic metastasis and multiple myeloma. Outcomes sought included pain relief, functional capacity, quality of life, vertebral height, kyphotic angle and adverse events. Studies were assessed for methodological bias, and estimates of effect were calculated using a random-effects model. Potential reasons for heterogeneity were explored.

**Results:**

The literature search revealed seven relevant studies published from 2003 to 2008, none of which were randomized trials. Analysis of those studies indicated that BKP resulted in less pain and better functional outcomes, and that these effects were maintained up to 2 years post-procedure. While BKP also improved early vertebral height loss and spinal deformity, these effects were not long-term. No serious procedure-related complications were described. Clinically asymptomatic cement leakage occurred in 6% of all treated levels, and new vertebral fractures in 10% of patients. While there is a lack of studies comparing BKP to other interventions, some data suggested that BKP provided similar pain relief as vertebroplasty and a lower cement leakage rate.

**Conclusion:**

It appears that there is level III evidence showing BKP is a well-tolerated, relatively safe and effective technique that provides early pain relief and improved functional outcomes in patients with painful neoplastic spinal fractures. BKP also provided long-term benefits in terms of pain and disability. However, the methodological quality of the original studies prevents definitive conclusions being drawn. Further investigation into the use of BKP for spinal fractures in cancer patients is warranted.

## Background

Spinal fractures are a common source of morbidity in patients with osteolytic metastasis and multiple myeloma [1-3]. Located principally in the thoracic and lumbar spine [4,5], these fractures often result in intractable back pain and impaired mobility because of vertebral height loss and spinal deformity [2,3,6]. In addition, the severe physiological and functional consequences have negative impacts on physical function, pulmonary capacity, nutritional state, psychological well-being and quality of life. Furthermore, spinal fractures increase the risk of new fractures, the number of hospitalizations and the incidence of all-cause mortality [1,3,6-8].

Traditional medical and surgical options often prove inadequate in spinal fracture patients. Due to the fragility and comorbidities associated with these patients, the surgical risk is high and open surgery is reserved only for cases with neurological involvement [1,5,6]. Additionally, non-surgical supportive treatments (e.g., analgesics, bed rest, use of braces or other external support systems, radiotherapy, hormone therapy, chemotherapy, radiopharmaceuticals and bisphosphonates) show variable outcomes, and single modality approaches are rarely effective [1,6,9,10].

In recent years, minimally invasive surgical techniques have emerged as an attractive option that reduce recovery time and surgical risks [1,5,11,12]. Balloon Kyphoplasty (BKP) is a percutaneous procedure used to relieve pain, restore vertebral height and reduce biomechanical alterations of the spine caused by fractures, and in turn improve physiological and functional outcomes [12-14]. BKP involves the introduction of a cannula into the vertebral body under image guidance, followed by the insertion of an inflatable bone tamp which is used to elevate the endplates. This reduces the deformity and creates a cavity within the vertebral body, and this cavity is subsequently filled with bone cement (polymethyl methacrylate, PMMA) in a controlled manner so as to minimize the risk of cement leakage [12-14].

Several reviews have recently shown that BKP is a relatively effective and safe treatment for painful osteoporotic vertebral fractures, its most common indication [12,15,16]. However, to our knowledge, BKP use in cancer patients has not been specifically analyzed.

The present study performed a meta-analysis of published reports describing the use of BKP in patients with spinal fractures of malignant origin. The study examined patient outcome data in order to determine the efficacy and safety of using BKP for spinal fractures in cancer patients.

## Methods

A systematic literature search was carried out up to September 2008 using several databases (MEDLINE, EMBASE, CINAHL, ISI Proceedings, The Cochrane Library, DARE, NHS EED and the HTA Database of the CRD). The search strategy was: #1: (balloon kyphoplasty), #2: (fracture*) or (vertebra*) or (neoplasm*) or (tumor*), #3: #1 and #2. There were no language restrictions. The search was completed manually using references from identified studies and reviews [17], and contact was made with experts in the field. No contact was made with industry.

### Inclusion criteria

Sackett's criteria [18], duly amended, were applied as follows: 1) population: studies conducted on more than 10 adults with spinal fractures of malignant origin; 2) intervention: BKP; 3) comparator: any medical or surgical treatment; 4) results: including a description of clinical outcomes regarding at least one of the following variables: pain, functional capacity, quality of life, vertebral height, kyphotic angle, cement leakages, clinical complications and new vertebral fractures.

No limitations were placed on study design or duration of follow-up [17].

### Selection of studies

The located studies were examined by two independent reviewers, and any disagreements were settled by discussion of the respective study data. During the data screening and extraction process, reviewers were not blinded to authors, institutions, or journals.

### Data extraction

Original data were extracted on a standard form that included details of the study design, information on the study population, and information on efficacy and safety outcomes.

### Analysis of methodological quality and scientific evidence

This was conducted in accordance with validated recommendations [19]. The possibility of bias in the studies was evaluated using published guidelines for systematic reviews [20].

### Data analysis and synthesis of results

To obtain an overall measure of the efficacy and safety of BKP, standard meta-analytical techniques were applied using the SE-STATA 9 computer software package (StataCorp LP Texas USA 1984-2007). Meta-analysis was conducted using a random-effects model [21]. Dichotomous outcomes were analyzed using rate ratios (RR) and corresponding 95% confidence interval. Continuous variables were analyzed using standardized or weighted differences in means (with 95% confidence intervals) between pre- and post- treatment values at the respective assessment dates. When an original study failed to provide a standard deviation of a continuous variable, it was estimated from the publication data (range or P-value) [22]. When the original study provided a standard error rather than a standard deviation, the latter was calculated using standard formulas. The degree of inconsistency across studies was evaluated using I^2 ^statistics, considering a value > 50% to be relevant [20]. We used sensitivity analysis to explore statistical heterogeneity. Results were deemed significant at a P-value < 0.05. In accordance with recent publications, funnel-plots were not used to estimate possible publication bias [23].

## Results

**Figure 1:**
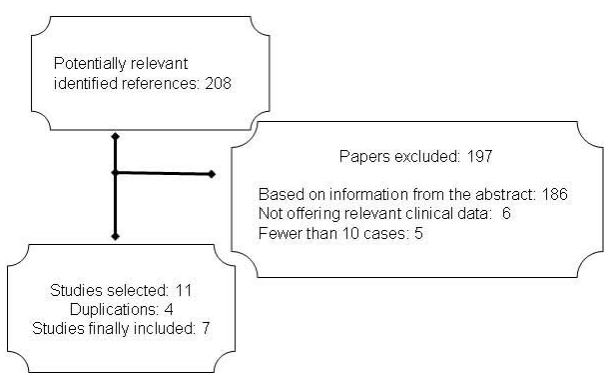
**Study selection and inclusion process**.

The literature selection process is summarized in Figure 1. After excluding references without an abstract and redundancies arising from the use of several databases, 208 potentially relevant references were identified. Eleven of these publications [24-34] were selected based on our inclusion criteria. Of those, 4 were excluded [24,29,31,32] because of data duplication in subsequent or more complete publications [25,33,34]. Hand searching of retrieved articles yielded no additional studies to be included.

The seven remaining studies were the basis of the present meta-analysis. These studies comprised three retrospective [26,28,30] and four prospective [25,27,33,34] single-center clinical series published from 2003 to 2008, and included data on 306 patients with 741 treated levels. The principal characteristics and quality assessment of these studies are summarized in Tables 1 and 2. The patients had a mean age of 62 years, 51% were male and 57% had multiple myeloma. The population mostly comprised patients with persistent pain secondary to thoracic or lumbar collapses, despite painkillers and medical therapy [25,27,30,34]. Around 30% of patients required devices to assist with walking. In all studies, symptomatic levels were identified by correlating the clinical data with MRI findings of marrow signal changes. In all but Kose's study [30], BKP was performed under general anesthesia. The mean number of spinal levels treated varied among the studies (range:1-6). Follow-up periods varied from 3 months [27] to 2 years [33,34].

**Table 1:** Principal characteristics of the included studies

**Author/year/** **country**	**Design**	**No. of patients/** **levels**	**Inclusion criteria**	**Exclusion criteria**	**Estimated age of VF**	**Follow-up**
Lieberman [25] 2003. USA	P	63/264	Painful progressive osteolytic VF secondary to multiple myeloma	Unstable (by virtue of myelomatous destruction of the posterior elements) or with retropulsed tissue or bone fragments.	11 mo (0.5-24 mo)	4 mo

Fourney [26] 2003. USA	R	BKP:15/32 VP: 34/65	VF with disabling pain refractory to prior medical and/or physical therapy in cancer patients	Epidural compression of the neural elements; failure to localize symptomatic levels; radicular pain; intolerance to being positioned prone or significant medical contraindications	3.2 mo (1 wk-26 mo)	4.5 mo

Lane [27] 2004. USA	P	19/46	Painful VF secondary to multiple myeloma	Not reported	>3 mo	3 mo

Vrionis [28] 2005. USA	R	50/128	VF with intractable mechanical pain refractory to medical and/or physical therapy in cancer patients.	Overt instability; clinical and/or radiological spinal cord compression; lesions above T3; absence of correlating symptoms (not mechanical pain and/or not localized to the area of VF).	13 cases:3 mo 35 cases: 10 mo	9 mo

Kose [30] 2006. Turkey	R	BKP: 18/22 VP: 16/26	Symptomatic VF in myeloma with pain refractory to medical therapy.	Canal stenosis	Not reported	12 mo

Pflugmacher[33] 2007. Germany	P	26/59	VF with severe refractory pain in patients with myeloma	Not reported	Not reported	24 mo

Pflugmacher[34] 2008. Germany	P	65/99	Metastatic VF with severe and refractory back pain.	Not reported	Not reported	24 mo

P: Prospective, R: Retrospective, BKP: Balloon Kyphoplasty, VP: Vertebroplasty, VF: Vertebral Fracture

**Table 2:** Quality assessment of included studies

	**Selection Bias**		**Procedure Bias**		**Detection Bias**		**Attrition Bias**
**Author/year**	**Representative sample**	**Consecutive cases**	**Specific Co-intervention**	**Pre-post assessment**	**Independent/** **blind assessment**	**Objective Results**	**Follow-up >80%**

Lieberman[25] 2003.	Yes	Yes	No	Yes	Yes	Yes	NR

Fourney[26] 2003.	Yes	Yes	Yes	Yes	Yes	Yes	No

Lane[27] 2004.	Yes	Yes	Yes	Yes	NR	Yes	Yes

Vrionis[28] 2005.	Yes	Yes	Yes	Yes	NR	NR	Yes

Kose[30] 2006.	Yes	NR	Yes	Yes	NR	Yes	Yes

Pflugmacher[33] 2007.	Yes	NR	Yes	Yes	NR	Yes	Yes

Pflugmacher[34] 2008.	Yes	Yes	Yes	Yes	Yes	Yes	No

NR: Not reported

The studies were examined for bias in accordance with validated references [19,20]. We found that most studies were designed to avoid most types of bias, with some exceptions (Table 2). However, a number of studies did not provide information on whether the results were assessed independently (Table 2). No study was randomized and the level of evidence corresponded to grade III [19]. However, the samples were representative and the studies were found to provide effective information on pre- and post-intervention variables and evaluated objective outcomes, and more than half reported follow-up of over 80% of patients [20].

### Efficacy outcomes

#### Pain Relief

The six studies that analyzed this variable before and after BKP reported reductions in pain intensity, and that the reduction was maintained during follow-up [see Additional file 1]. Combined analysis of studies that contributed Visual Analog Scale (VAS) data showed that BKP resulted in a reduction in mean pain score both in the postoperative period and at the end of the follow-up period (Table 3). A substantial level of inconsistency was found across studies, with the mean pre-procedure VAS being related to the effect size.

**Table 3:** Efficacy of BKP for malignant spinal fractures: Results of meta-analysis

**Variable**	**Studies providing data**	**N° patients/levels**	**Size of effect (95%CI); P-value; I**^**2**^
**Pain: VAS score (0-10)**			
Basal-postoperative	4 [25,30,33,34]	172 patients	SMD: 3.85 (2.99, 4.71); p < .001; 79%
Baseline-end of follow-up	3 [30,33,34]	109 patients	SMD: 4.27 (2.38, 6.21); p < .001; 93%

**Functional capacity:**			
**ODI (0-100)**			
Baseline-postoperative	4 [25,27,33,34]	173 patients	WMD:-28.78 (-11.5,- 46.0);p = .001; 99%
Baseline-<6 months	2 [25,27]	82 patients	WMD:-16.39 (-14.25,-18.5);p = .001; 0%
Baseline-2 years	2 [33,34]	91 patients	WMD:-41.95 (-39.42, -44.5);p = .001; 0%

**Kyphotic deformity (Cobb angle):**			
Basal-postoperative	3 [26,33,34]	180 levels	SMD:-0.69 (-0.20, -1.16); p = .001; 78%
Baseline-end of follow-up	3 [26,33,34]	155 levels	SMD: -0.39 (0.05, -0.84); p = .08; 74%

**Vertebral height:**			
**% of restitution**	3 [25,26,30]	342 levels	RR:47% (33%, 61%); 38%
**Increase (mm):**	2 [33,34]	158 levels	
**Anterior vertebral body**			
Basal-postoperative			SMD:0.28 (0.06, 0.51); p = .01; 0%
Baseline-end of follow-up			SMD: 0.15 (-0.16, 0.45); p = .35; 37%
**Midline vertebral body**			
Basal-postoperative			SMD:0.28 (0.003, 0.56); p = .04; 34%
Baseline-end of follow-up			SMD:0.15 (-0.17, 0.46); p = .35; 41%

VAS: Visual Analog Scale. SMD: Standardized mean difference ODI: Oswestry Disability Index. WMD: Weighted mean difference. RR: rate ratio. CI: Confidence Interval. All based on a random effects meta-analysis.

Fourney's study [26] showed that both BKP and vertebroplasty relieved pain to a similar degree in a high percentage of patients. Kose's study [30] indicated that although both BKP and vertebroplasty relieved pain, BKP provided greater pain relief at 6 and 12 months postoperatively [see Additional file 1].

#### Functional Capacity

Changes in functional capacity were recorded in four studies using the validated Oswestry Disability Index (ODI 0-100) [25,27,33,34]. In all studies, comparisons between preoperative and postoperative values showed a significant decrease in ODI scores after treatment, indicating a decrease in impairment [see Additional file 1]. The combined analysis indicated improved functional capacity after BKP, and that the improvement was sustained over the follow-up period (Table 3). Heterogeneity was found to be related to differences in the mean basal pre-procedure ODI scores.

Individual studies [26,28,30] reported that most patients were mobilized on the same day following the procedure and regained activity, social functions and physical capacity.

#### Quality of Life

All studies reported obvious improvement in patient quality of life after the procedure. However, only one study [25] evaluated the effect on quality of life using the SF-36 questionnaire. That study found significant improvements in physical function, physical role, bodily pain, vitality, social functioning, and mental health, but no improvements in general health perception or emotional role [see Additional file 1].

#### Kyphotic Deformity

Three studies analyzed this variable using the absolute Cobb angle value [26,32,33]. Joint analysis in these studies found that BKP resulted in a decrease in the angle's absolute value (Table 3), although with a high degree of heterogeneity and a wide variation among studies in both baseline and post-treatment values of deformity [see Additional file 1]. Follow-up analysis showed partial loss of the initial effect, with the absolute value of the angle ultimately decreasing to preoperative levels (Table 2) and the pooled differences being not significant (Table 3).

#### Vertebral Height

Although the number of levels varied among studies, each study in which this variable was recorded [25-27,30,33,34] reported a post-BKP increase in vertebral height. However, the increase was expressed differently in each study [see Additional file 1]. Several authors [25-27] recorded the percentage of vertebral height restoration after BKP, and the mean restoration across these studies was 47% (Table 3). In contrast, Pflugmacher [33,34] measured vertebral height gained in millimeters. Pooled analysis of his data showed increases in both anterior and midline vertebral body after BKP. However, neither of these increases was statistically maintained at the end of the follow-up period.

### Safety

#### Cement Leaks

**Figure 2:**
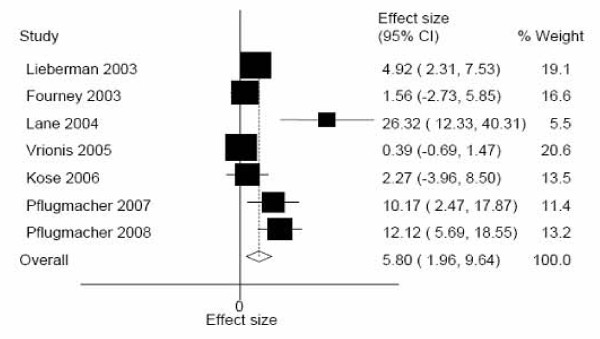
**Balloon Kyphoplasty: Meta-analysis of cement leakage**. Random-effects meta-analysis. CI: Confidence interval.

All studies provided safety information. Overall, there were 41 cement leakages associated with BKP, none of which was symptomatic. BKP cement leakages occurred at a mean of approximately 6% across all levels (Figure 2). Sensitivity analysis showed that inconsistency across studies (I^2^: 84%) was related to design, with prospective studies yielding a higher rate of leakage (11.2%) than retrospective studies (0.51%, I^2^: 0%). No relationship was found with other factors such as specific etiology (multiple myeloma vs. metastasis) or estimated age of the fracture.

In Fourney's study [26], 6 asymptomatic leakages were observed in the 65 levels treated with vertebroplasty, representing 9%, while no leakages were observed in patients treated with BKP. Kose's comparative study [30] recorded no cement leaks in patients treated with either BKP or vertebroplasty.

#### New Vertebral Fractures

**Figure 3:**
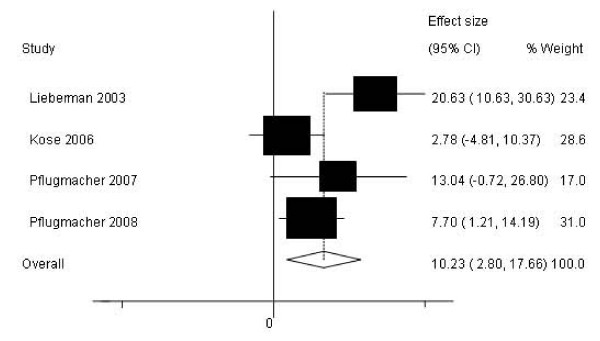
**Balloon Kyphoplasty: Meta-analysis on the incidence of new spinal fractures**. Random-effects model. CI: Confidence interval.

After pre-planned radiographic evaluation during follow-up, four studies reported the development of 21 new vertebral fractures in 172 patients [see Additional file 1]. Although fracture rates varied widely among studies, pooled analysis showed an overall rate close to 10% (Figure 3). Sensitivity analysis showed that inconsistency across studies (I^2^: 54%) was related to fracture etiology. The rate of new fractures was higher in patients with myeloma (12.4%) than in those with metastasis (7.9%, I^2^:0%).

#### Clinical Complications

Although poorly reported across the studies, a small number of patients treated with BKP experienced clinical complications, but no deaths were reported within 30 days of BKP [see Additional file 1]. Two studies reported complications unrelated to the procedure. In Fourney's study [26], one patient was readmitted to hospital 15 days after BKP due to an exacerbation of pre-existing congestive heart failure, while Vrionis et al. [28] recorded a case of asystole in a patient with a history of lung cancer with multiple brain metastases. No evidence of pulmonary embolism due to cement leakage was seen postoperatively and the exact cause of asystole was undetermined. That patient recovered to her preoperative level, but died 1 month after surgery from unrelated causes.

In a comparative study, Kose [30] recorded no post-surgical neurological or pulmonary complications after any intervention. Although that article stated that two patients suffered minor clinical complications (wound infection and temporary respiratory difficulties while being placed in position for surgery), the authors did not indicate which intervention had been performed (i.e., BKP or vertebroplasty).

## Discussion

The present study found that there are very few reports on the efficacy and safety of BKP for treating tumor-associated spinal fractures. Furthermore, all such reports have a non-randomized design, and are limited in terms of number of patients, procedures and reported outcomes. Nonetheless, a combined analysis of these reports provided results in broad agreement with earlier reports examining osteoporotic fractures [15,16,34-37].

The present meta-analysis found that BKP provided immediate pain relief, and that the relief can continue for up to 2 years. This pain relief is not only clinically significant (i.e., a change of 2.0 - 2.7 points on the VAS, which is equivalent to a reduction of 30 - 41%) [38], but of great benefit because most patients have intense and refractory pain resistant to painkillers and conventional medical therapy. The mechanism underlying this pain relief remains to be identified [11].

The current study also found that BKP resulted in improved functional outcomes. BKP improved functional capacity as assessed by the Oswestry Disability Index. In addition, the study that assessed quality of life using the standard SF-36 questionnaire found improvement in nearly every domain, including vitality, social function and mental health. Interestingly, that study found no significant improvement in general health perception, which was not clearly explained but may reflect progression of the primary malignant disease.

The third major finding of this study was that BKP appeared to reduce the kyphotic angle and, at least partially, restored the height of the collapsed vertebral body. However, this evidence was limited due to the diverse methods used for these assessments. In addition, there was a progressive postoperative decrease in the amount of improvement, and the morphologic benefits were not maintained over the entire follow-up period. Hence, these findings do not suggest that the technique prevents the severe physiological and systemic effects of spinal fractures, which is one of its main objectives [13,14].

Regarding safety, it appears that BKP is a safe procedure. Though BKP was usually performed under general anesthesia, very few clinical complications were reported, of which none was serious. No study recorded neurological or pulmonary complications. Most studies found that any clinical complications were not directly related to the technique but to comorbidities or the progression of the initial disease. However, given the typical frailty of BKP patients, the procedure should be carried out in centers equipped to treat possible neurological or cardiopulmonary complications.

Cement leakages occurred in approximately 6% of all treated levels. While study design influenced that analysis, leakages were asymptomatic in all cases. Our results indicate that approximately 10% of patients will develop a new vertebral collapse within two years after treatment. The development of new vertebral fractures seems to be more frequent (nearly double) in patients with multiple myeloma than in metastatic cases. This is not surprising given several studies have found myeloma patients are more vulnerable to fractures [2]. The present figures compare favorably with other published data [15,16,35]. Overall, the data suggest that the incidence of new fractures after a BKP-based intervention is not higher than the spontaneous incidence of new vertebral fractures (19-24%) described for untreated patients [8].

Finally, mention should be made to the fact that, at this time, there is an evident lack of studies comparing BKP with other interventions, both invasive and non-invasive. The present findings indicate that BKP and vertebroplasty provide similar pain relief, but that BKP is associated with a lower cement leakage rate, as has been observed elsewhere [35-37].

The current study has several potential limitations. Publication bias may exist by limiting our search to peer-reviewed literature. Nevertheless, we feel that any such bias would have been minimized by the scope of and systematic strategy used in the search of the literature, and we are confident that most research conducted in this field was identified [17,39]. We did not include unpublished data from industry given both the difficulties encountered in obtaining such information and the recognition that the use of such data may not necessarily reduce the bias in a meta-analysis [39,40].

The methodological quality of the studies, particularly their non-randomized design, may constitute another limitation of this work [20]. To our knowledge, there are no published reports of randomized clinical trials examining the use of BKP for malignant spinal fractures. However, such an absence should not prevent analysis of its efficacy and safety, and evaluation of new technologies and procedures, as this would deprive patients, professionals and health authorities of essential information for decision-making [41-44]. The present study followed the guidelines outlined by the Meta-analysis of Observational Studies in Epidemiology Group [43] in order to identify and collate relevant data from available studies.

Lastly, we should recognize that the present findings were based on original studies involving relatively small sample sizes, making them susceptible to the inherent problems associated with such a study design [45].

## Conclusion

The present study found that there is level III evidence showing that BKP is a well-tolerated, relatively safe and effective method for reducing pain and improving functional outcomes in patients with painful neoplastic spinal fractures. While BKP also provided immediate improvement in vertebral height loss and spinal deformity, these morphologic changes were not long-term. The limited data available in this area indicate the need for long-term high-quality controlled studies to ascertain the clinical role of BKP for patients with painful neoplastic spinal fractures. Future studies should also have consistent reporting of clinically useful outcomes.

## Competing interests

CB and JMA have received prior grant support from Kyphon Iberica SL (to Instituto de Salud Carlos III). No other potential competing interests relevant to this article exist. No corporate/industry funds were received in support of this work. None of the authors has a financial interest in any of the devices discussed in this report.

## Authors' contributions

CB conceived the study, participated in its design, performed statistical analysis and drafted the manuscript. TL participated in the design of the study and performed statistical analysis. PC participated in acquisition, analysis and interpretation of data and helped draft the manuscript. ZS participated in acquisition, analysis and interpretation of data, and drafted the manuscript. JMA conceived the study and participated in design and coordination. All authors read and approved the final manuscript.

## Pre-publication history

The pre-publication history for this paper can be accessed here:



## Supplementary Material

Additional file 1 **Efficacy of BKP for malignant spinal fractures: Individual description of outcomes**. The data provided represent the individual studies description of outcomes. Click here for file
