# Supplementary material for: Balloon kyphoplasty in malignant spinal fractures: a systematic review and meta-analysis
Source: BMC Palliat Care. 2009 Sep 9;8:12. doi: 10.1186/1472-684X-8-12 (PMC2746801; doi:10.1186/1472-684X-8-12)
Supplement: Additional file 1 — Efficacy of BKP for malignant spinal fractures: Individual description of outcomes. The data provided represent the individual studies description of outcomes. [file 1472-684X-8-12-S1.doc]

**Additional File. Efficacy of BKP for malignant spinal fractures: Individual description of outcomes**

| **Author/ year of publication** | **Pain relief** | **Functional capacity, ODI** (0-100). | **Quality of life. Pre/Post-BKP in SF-36 domains** (0-100) **mean value; p-value** | **Kyphotic angle**  **(grades).** | **Vertebral height** | **Cement Leakage** | **New VF** | **Clinical**  **complications** |
| --- | --- | --- | --- | --- | --- | --- | --- | --- |
| **Lieberman**^[25]^  2003 | 52 myeloma patients  VAS (0-10):  Pre 6.18 ; Post 2.84; p<.0001 | Baseline: 46.7  Postoperative: 30.3  Mean change:-16.3; p<.0001 | Physical function: 25/47; p<.001  Physical role: 11/27; p=.04  Bodily pain: 28/48; p=.0003  Vitality: 33/ 41; p=.043  Social function: 47/63; p=.016  Mental health: 69/77; p=.008  Emotional role: 62/60; p=.80  General health:47/48; p=.68 | NR | Mean restored: 34% (0-100%).  Mean increase: 2.9 mm | <5% asymptomatic | 21% | None |
| **Fourney**^[26]^  2003 | BKP:  Complete 7%  Improvement 73%  VP:  Complete 23%  Improvement 63%  NS | NR | NR | Pre: 25.7±9.7  Post: 20.5±8.7 p=.001  Improvement: 4.1±3.72 | Height lost: 9.7±5.1mm  Restored height: 4.5±3.6 mm; p=.01  Restoration: 42±21% | BKP: 0  VP: 9.2%  asymptomatic | NR | No related complications |
| **Lane**^[27]^  2004 | NR | Improvement in 84% of patients.  Baseline: 48.9±17  Final: 32.6±13.6; p<.001 | NR | NR | Anterior vertebral height restituted in 76% of levels. Mean + SD: 37.8±26.9% p<.01  Middle vertebral height restituted in 91% of levels.  Mean + SD: 53.37±29% p<.001 | 10 asymptomatic | NR | None |
| **Vrionis**^[28]^  2005 | Improvement in 96% of patients.  No pain in 20% of patients | NR | NR | NR | NR | None | NR | 1 non-related asystole |
| **Kose**^[30]^  2006 | VAS (0-10)  BKP vs. VP:  6 wk: p=.106  6 mo: p=.024  1 yr: p=.027 | NR | NR | NR | Mean restoration: 54% (25-72) | BKP: none  VP: none | BKP: none  VP: none | 1 balloon rupture, 1 respiratory distress,  1 wound infection |
| **Pflugmacher**^[33]^  2007 | VAS (0-10)  Pre-BKP: 8.6±2.7  Post: 2.4±1.6; p<.05  3 mo: 1.8±0.9;p<.05  6 mo :2.2±1.1;p<.05  1 yr: 2.5±1.3; p<.05  2 yr: 3.1±1.8; p<.05 | Pre-BKP: 78.1±27.3  Post: 37.3±14.1; p<.05  3 mo: 27.2±9.4; p<.05  6 mo: 28.2±10.2; p<.05  1 yr: 28.8±11.6; p<.05  2 yr: 33.6±13.3; p<.05 | NR | Pre-BKP:16 ±8  Post: 8±6; p<.05  3 mo: 9±6; p<.05  6 mo: 9±6; p<.05  1 yr: 10±6.5; p<.05  2 yr: 11±7; p<.05 | Improvement in 64.5% of fractured bodies.  Anterior Region, mm  increase: 3.8 mm (0-16)  Middle Region, mm  increase:4.3 (0- 17.5) | 6 asymptomatic | 13% | None |
| **Pflugmacher**^[34]^  2008 | VAS (0-10)  Pre-BKP: 8.3±1.5  Post: 3.3±0.9;p<.001  3mo: 2.9±0.9; p<.001  6 mo: 2.9±1; p<.001  1 yr: 3.1±1; p<.001  2 yr: 3.2±1; p<.001 | Pre-BKP: 81±8  Post: 39±7; p<.001  3 mo: 33±6; p<.001  6 mo: 32±7; p<.001  1 yr: 32±7; p<.001  2 yr: 35±6; p<.001 | NR | Pre:12±2.3  Post: 11.1±2.4;  p<.001 vs. pre.  3 mo: 11.5±2.4;  p<.001 vs. pre  6 mo: 11.8±2.4;  p<.05 vs. pre  1 yr: 11.8±2.4;  p<.05 vs pre  2 yr: 12±3.3;  NS vs. pre | Anterior Region, mm  Pre: 23.7±2.4  Post: 24.2±2.4; p<.001  3 mo: 24.2±2.5; p<.001  6 mo: 23.9±25; p<.001  1 yr: 23.6±2.4; p<.05  2 yr: 23.7±2.3; p<.05  Midline Region, mm  Pre: 24.5±1.8  Post: 24.8±1.9; p<.001  3 mo: 24.8±1.9; p<.05  6 mo: 24.6±1.9; p<.05  1 yr: 24.5±1.9; p<.001  2 yr: 24.5±1.9; NS | 12 levels (12.5%) asymptomatic | 5 patients (8%)  adjacent | None |

 BKP: Balloon kyphoplasty. VP: Vertebroplasty. New VF: New vertebral fracture. ODI: Oswestry Disability Index. NR: Not recorded. NS: Non significant. Individual results are shown as reported in the original publications. Continuous data are expressed as mean or mean±SD.
